# Mechanism exploration of Gouqi-wentang formula against type 2 diabetes mellitus by phytochemistry and network pharmacology-based analysis and biological validation

**DOI:** 10.1186/s13020-021-00479-2

**Published:** 2021-09-27

**Authors:** Lin Han, Hao-yu Yang, Yu-jiao Zheng, Xiu-xiu Wei, Wen-chao Dan, Li-li Zhang, Qi-you Ding, Xu Ma, Xin-miao Wang, Lin-hua Zhao, Xiao-lin Tong

**Affiliations:** 1 Guang’anmen Hospital, China Academy of Chinese Medical Sciences, Beijing, 100053 China; 2 Beijing University of Chinese Medicine, Beijing, 100029 China; 3 Gansu University of Chinese Medicine, Lanzhou, 730000 China

**Keywords:** T2DM, Gouqi-wentang formula (GQWTF), LC–MS, Network pharmacology, Biological validation

## Abstract

**Background:**

The Gouqi-wentang formula (GQWTF) is a herbal formula used by Academician Xiao-lin Tong for the clinical treatment of T2DM. GQWTF is beneficial to qi, nourishes Yin, clears heat, and promotes fluid production, but the effective components and their mechanism of action remain unclear.

**Methods:**

The main components of GQWTF were detected by LC–MS, and the multi-target mechanisms of GQWTF in T2DM were elucidated using network pharmacology analysis, including target prediction, protein–protein interaction network construction and analysis, Gene Ontology (GO) terms, Kyoto Encyclopaedia of Genes and Genomes (KEGG) pathway annotation, and other network construction. Finally, the efficacy of the GQWTF was verified using biological experiments.

**Results:**

First, the “herb-channel tropism” network suggested that GQWTF focuses more on treating diseases by recuperating the liver, which is considered as an important insulin-sensitive organ. Subsequently, a total of 16 active ingredients in GQWTF were detected and screened, and their biological targets were predicted. Then, “compound-target” network was constructed, where enrichment analysis of GQWTF targets reflected its potential pharmacological activities. After T2DM-related target identification, 39 cross targets of GQWTF and T2DM were obtained, and 30 key targets highly responsible for the beneficial effect of GQWTF on T2DM were identified by PPI analysis. GO analysis of these key targets showed that many biological processes of GQWTF in treating T2DM are key in the occurrence and development of T2DM, including components related to inflammatory/immune response, insulin, and metabolism. KEGG analysis revealed the regulation of multiple signalling pathways, such as insulin resistance, PPAR signalling pathway, FoxO signalling pathway, Fc epsilon RI signalling pathway, and pathways that influence diabetes primarily by regulating metabolism as well as other T2DM directly related pathways. Furthermore, a “formula-compound-pathway-symptom” network was constructed to represent a global view of GQWTF in the treatment of T2DM.

**Conclusions:**

This study explored the mechanism of action of GQWTF in T2DM by multi-component and multi-target multi pathways, which could provide a theoretical basis for the development and clinical application of GQWTF.

**Supplementary Information:**

The online version contains supplementary material available at 10.1186/s13020-021-00479-2.

## Background

With about 463 million adults suffering from diabetes mellitus worldwide, the disease has become a serious global public health concern. Type 2 diabetes mellitus (T2DM) accounts for up to 90% of the total number of these patients [1]. Epidemiological studies have shown that obesity, a high-calorie diet, and lack of physical activity are high risk factors for T2DM [2], suggesting a close correlation between high-calorie diet and T2DM [3]. With the improvement of people's living standards, the phenomenon of a high-calorie diet and inadequate physical activity will persist, and the number of people with T2DM worldwide is estimated to rise to 630 million by 2045 [4], prevention and control of T2DM is required.

Modern medical treatment of T2DM mainly focuses on the control of blood sugar by oral hypoglycemic drugs and injection of insulin, whereas improper use of insulin can lead to hypoglycemia [5]. Traditional Chinese medicine (TCM) has been in use for treating T2DM for 2000 years, which can improve the clinical symptoms, delay islet β-cell failure, improve insulin resistance, and avoid or reduce the use of glucose drugs. It improves the quality of the life of patients, and TCM offers a safe and effective alternative for the treatment of diabetes [6–8]. The Gouqi-wentang formula (GQWTF) was derived from Academician Tong Xiaolin's experience in the prevention and treatment of T2DM for many years. It consists of Gou-Qi-Zi (GQZ, Lych fructus), Sang-Ye (SY, Mori folium), Zhi-Mu (ZM, Anemarrhenae rhizome), Chi-Shao (CS, Radix paeoniae rubra), and Xi-Yang-Shen (XYS, *Panax quinquefolium*). The five herbs in the formula are combined to benefit the qi and nourish yin, which clear the heat and promote fluid production. Pharmacological studies have shown that the herbs in GQWTF could reduce blood sugar and cut off the progress of the disease, for example, polysaccharides extracted from GQZ have hypoglycemic and hypolipidemic effects [9]. Water extracts of SY can reduce the blood glucose of diabetic mice and improve glucose tolerance [10]. ZM extract significantly reduces fasting blood glucose and markedly increase the size and the number of insulin-producing β-cells in mice by mediating activation of AMPK [11]. A double-blind, randomized, cross-over clinical trial indicates that XYS can attenuate fasting blood glucose, reduce blood glucose, and ameliorate glucose metabolism [12]; whereas CS ethanol extract plays a role in multiple hypoglycemic bioactivities in vivo by transcriptional inhibition of gluconeogenesis [13]. Due to the multiple components and multiple targeting characteristics of Chinese herbal medicine, studies aimed at single herbs are not enough to clarify the underlying mechanism of GQWTF. Therefore, it is necessary to resort to a new form of the TCM research system.

Network pharmacology provides an opportunity for research on TCM mechanisms, which are based on systems biology and network biology theory [14]. It analyses the relationship between drugs and diseases by constructing more reliable molecular networks associated with drug action targets and molecular networks of related biological entities. Thus, it can reflect specific disease states more realistically, integrate drug action target networks with disease biological networks, analyse drug-target-disease networks, and predict key targets. Therefore, network pharmacology can comprehensively analyse the effects of drugs on the human body from a holistic perspective, which is consistent with the "holistic" concept of TCM [15].

**Fig. 1:**
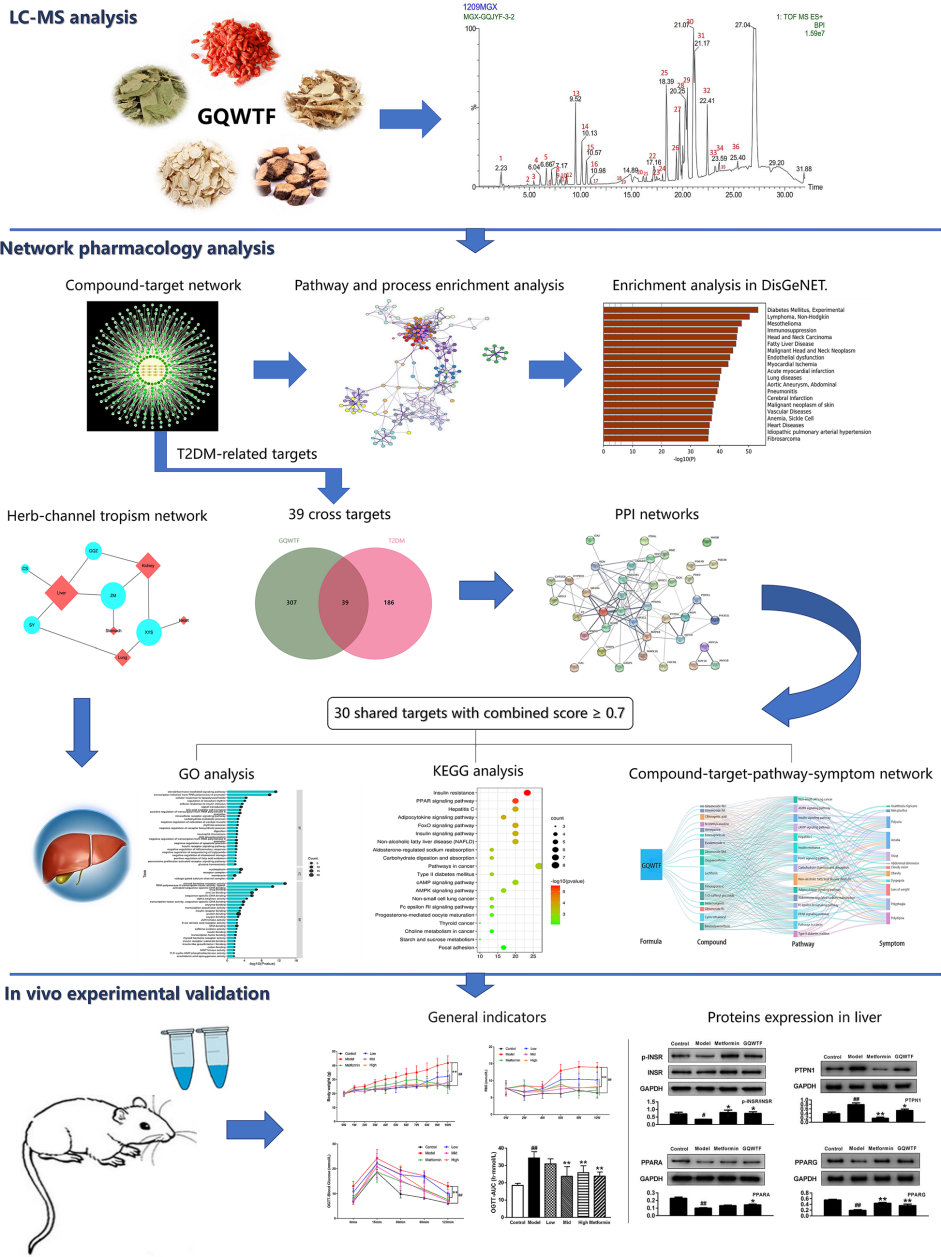
Workflow of this study

In this study, we implemented a systematic approach to explore the pharmacological mechanisms of GQWTF in the treatment of T2DM. The study was divided into two parts. The first part consisted of network pharmacology analysis. The ingredients of GQWTF were identified by liquid chromatography coupled with mass spectrometry (LC–MS). The final list of bioactive ingredients of GQWTF was selected based on existing research. Network pharmacology analysis predicted the bioactive ingredients’ targeted genes/signalling pathways involved in T2DM. The second part consisted of animal experiments. The hypoglycemic effect of GQWTF was first validated in T2DM C57BL/6J mice fed with a high-fat diet. The predicted targets/pathways were further validated using samples from T2DM mice treated with GQWTF. In summary, we investigated the therapeutic mechanisms of GQWTF against T2DM for the first time by network pharmacology and experimental validation. Figure 1 shows the flow chart of this whole analysis.

## Methods

### Collection of GQWTF meridian

According to TCM theory, meridian tropism can reflect the organ in which TCM can exert its curative effect. By searching Chinese Pharmacopoeia 2020 edition (National Medical Products Administration, China), we confirmed the TCM meridians of the herbs in GQWTF, and we imported the information into Cytoscape3.7.0 to construct the herb-meridian network.

### Detection and screening of the active components of GQWTF

The prepared liquid medicine was filtered through a 0.22 μm filter membrane, and the liquid medicine was filled into a clean vial and half-stoppered before freezing to solid ice at an ultra-low temperature. The vial filled with liquid medicine was placed in a vacuum freeze dryer at an ultra-low temperature. Then, it is placed in a vacuum so that the water in the solid ice sublimation is directly excluded, thus forming the freeze-dried powder. The precisely weighed freeze-dried powder of GQWTF was placed in a 10 mL volumetric flask and dissolved in 8 mL of 50% methanol. The flask was then sealed and sonicated in a KQ-300 ultrasonic water bath (Kunshan Ultrasonic Instrument Co. Ltd., Jiangsu, China) operating at 40 kHz with an output power of 300 W for 30 min at room temperature. After standing and cooling, 10 mL methanol/water (50:50, v/v) was added and mixed well. The extract was filtered through a 0.22 μm microporous membrane and stored at 4 °C in the dark until analysis. Samples were analysed on a Waters Synapt High-Definition MS System (Waters Corporation, Milford, MA, USA) equipped with a Waters BEH Phenyl column (1.7 µm, 2.1 × 100 mm). The analytical column was maintained at 35 °C and the mobile phase consisted of water containing 0.1% formic acid (A) and acetonitrile (B). The gradient running procedure was programmed as follows: 0–4 min, 95% A; 4–5 min, 95–90% A; 5–10 min, 90–88% A; 10–14 min, 88–75% A; 14–20 min, 75–63% A; 20–23 min, 63–50% A; 23–25 min, 50–10% A; 25–30 min, 10%–0 A; and 30–35 min, 0–95% A. The injection volume was 5 μL and the flow rate was 0.2 mL/min.

For mass spectrometry conditions, the cone voltage was set at 40 V in the negative ion mode, and the capillary voltage was set at 2500 V. We used a desolvation temperature of 400 °C, source temperature of 100 °C, and extraction cone of 3 V. The desolvation gas flow rate was set at 800 L/h, the cone gas flow rate was set at 50 L/h, and the mass range was from m/z 100 to 1000. The data were collected and acquired using MassLynx V4.1 software (Waters Co., Milford, MA, USA). The components obtained by LC–MS analysis were screened by absorption, distribution, metabolism, and excretion (ADME) against related references. The inactive compounds were removed, and the remaining compounds were the potential active ingredients of GQWTF.

### Collection and screening of targets of compounds and disease

We retrieved the structures of the active ingredients of GQWTF on PubChem and downloaded their SDF files. Then, the SDF files were imported into PharmMapper [16] (http://lilab-ecust.cn/pharmmapper/) to predict the potential targets of these compounds. The parameters were set as follows: generate conformers: Yes; maximum generated conformations: 300; select targets set: human protein targets only (v2010, 2241); and number of reserved matched targets (Max 1000): 300. All predicted targets with “fit scores” ≥ 3 were retained. The UniProt IDs of the targets were introduced into the UniProt database (https://www.uniprot.org/uniprot/) with the properties set at “reviewed” and “human” to search for the official symbol of each target.

Targets related to T2DM were searched using the keywords “Type 2 diabetes mellitus” in DrugBank [17] (https://go.drugbank.com/) and Therapeutic Target Database (TTD) [18] (http://db.idrblab.net/ttd/). After combining the targets of the two databases and removing repetitive targets, we identified disease targets and sent them to the UniProt database for normalisation.

### Construction and analysis of compound-target network

The compound-target network was constructed for the 16 potential active ingredients of GQWTF and related targets using Cytoscape to interpret the pharmacological mechanisms of GQWTF in total, such as further observation of the biological functions of key components and targets.

For candidate targets of active ingredients in the GQWTF, pathway and process enrichment analyses were carried out using Metascape [19] with the following ontology sources: Kyoto Encyclopaedia of Genes and Genomes (KEGG) Pathway, Gene Ontology (GO), biological processes (BP), Reactome Gene Sets, Canonical Pathways, CORUM, TRRUST, DisGeNET [20] (https://www.disgenet.org/), PaGenBase, transcription factor targets, WikiPathways, PANTHER Pathway, and COVID. Terms with a *p*-value < 0.01, a minimum count of 3, and an enrichment factor > 1.5 were collected and used for filtering. The remaining significant terms were then hierarchically clustered into a tree based on the kappa statistical similarities among their gene memberships. A 0.3 kappa score was applied as the threshold to cast the tree into term clusters, and the most statistically significant term within a cluster was chosen to represent the cluster.

Furthermore, gene list enrichment was identified in the ontology categories of DisGeNET, and all genes in the genome were used as the enrichment background. Terms with a *p*-value < 0.01, a minimum count of 3, and an enrichment factor > 1.5 were collected and grouped into clusters based on their membership similarities.

### Protein‒protein interactions (PPI)

The overlapping targets of GQWTF and T2DM were taken by Venn diagram, and they were uploaded to the STRING database (http://string-db.org/) to obtain the PPI. With the species limited to “Homo sapiens”, we selected targets with “confidence score” > 0.7 for further enrichment analysis.

### Enrichment analysis

Enrichment analysis of key targets obtained from the PPI network analysis of candidate targets of GQWTF for the treatment of T2DM, including GO and KEGG pathway analysis, was performed using the DAVID 6.8 database (https://david.ncifcrf.gov/), with a threshold value of *p* < 0.05. GO analysis was performed to reveal the function of gene targets, including BP, molecular functions (MF), and cellular components (CC). Key signalling pathways of gene targets were obtained by KEGG analysis.

Furthermore, a compound-target-pathway-symptom network was constructed by connecting the active compounds, overlapping targets, pathways, and symptoms. Pathway-symptom relationships were obtained from the literature.

### Experimental design

Sixty healthy male C57BL/6J mice (7 weeks old) were purchased from the Model Animal Research Center of Nanjing University. Ten mice were housed in cages under specific pathogen-free grade (SPF grade) conditions. All mice were maintained under controlled conditions with a temperature of 22–25 °C, relative humidity of 55 ± 5%, a regular 12 h light/dark cycle (light time 7:00–19:00), and free access to food and water. All procedures were approved by the Institutional Animal Care and Use Committee, Guang'an men Hospital, China Academy of Chinese Medical Sciences (IACUC-GAMH-2021-004).

After 1 week of adaptive raising, mice were randomly divided into six groups with 10 mice per group: control group (sterile water), model group (sterile water), metformin group (metformin 250 mg/kg/d), low-dose group (1/2 human equivalent dose (HED) of GQWTF converted by "body specific surface area method"), medium-dose group (HED of GQWTF), and high-dose group (double HED of GQWTFD). During the 10-week experiment, except for the mice in the control group which were given normal feed, the other five groups received high-fat diet (60%) (D12492, Research Diets, New Brunswick, NJ, USA). Pharmacological intervention was started at the same time after grouping, and all pharmaceutical interventions were administered via gavage once a day.

During the experiment, body weight was measured once a week and fasting blood glucose (FBG) was measured every 2 weeks. After 10 weeks of treatment, an oral glucose tolerance test (OGTT) was performed at 0, 15, 30, 60, and 120 min after administration of 20% glucose at a dose of 2 g/kg. At the end of the experiment, all the mice were euthanized by cervical dislocation after a 12-h fasting period, and liver tissues were harvested and stored at − 80 °C for western blot analyses. The protein was isolated from the hepatic tissue by phenylmethylsulfonyl fluoride, and the protein concentration was measured using a bicinchoninic acid assay kit. These protein samples were separated by 10% SDS–polyacrylamide gel electrophoresis, and the proteins were electroblotted onto a polyvinylidene difluoride membrane. The membranes were blocked with 5% BSA for 1 h and incubated with the following primary antibodies at 4 °C overnight: rabbit anti-p-INSR (1:1000 dilution, ab60946, Abcam plc, UK), rabbit anti-INSR (1:1000 dilution, ab137747, Abcam plc, UK), rabbit anti-PTPN1 (1:1000 dilution, ab252928, Abcam plc, UK), rabbit anti-PPARA (1:800 dilution, ab24509, Abcam plc, UK), rabbit anti-PPARG (1:500 dilution, ab209350, Abcam plc, UK), and rabbit anti-GAPDH (10B8) (1:10,000 dilution, KGAA002, KeyGEN BioTECH, China). After washing with TBST, the membranes were incubated with horseradish peroxidase (HRP)-conjugated goat anti-rabbit IgG secondary antibody on a shaker at room temperature for 2 h. The antigen–antibody complexes were visualised using an enhanced chemiluminescence system. Relative protein expression levels of p-INSR, INSR, PTPN1, PPARA, and PPARG were normalised to that of GAPDH.

### Statistical analyses

Data are expressed as the mean ± standard deviation (SD) of at least three independent experiments. Statistical analyses were performed using the SPSS software (SPSS 20.0, SPSS Inc., Chicago, IL, USA). Two-tailed Student’s t-test, one-way analysis of variance (ANOVA) with Dunnett’s post-hoc tests, and Games-Howell tests were used to compare the groups. *p* < 0.05 was considered statistically significant.

## Results

### Channel tropism information of herbs in GQWTF

**Fig. 2:**
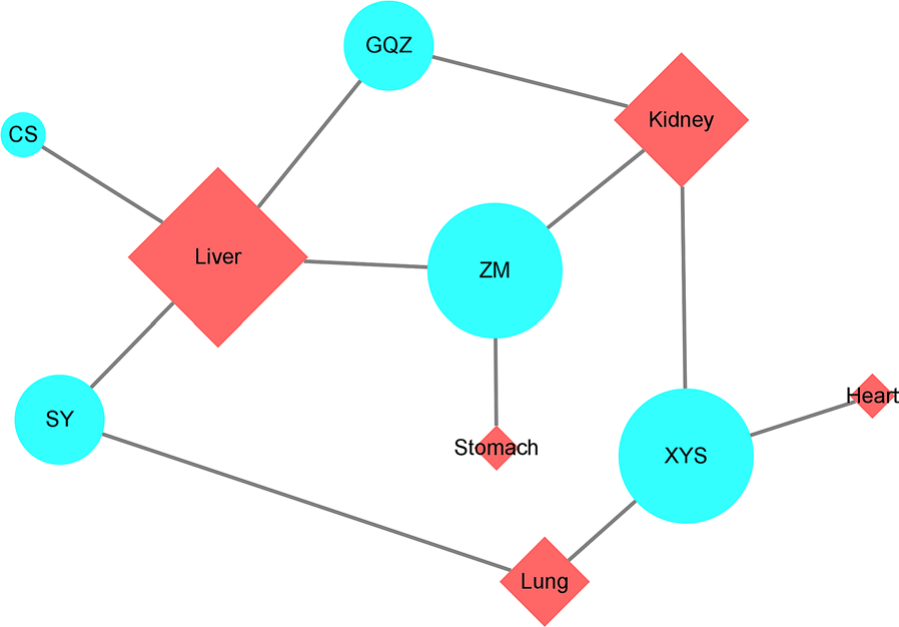
The “herb-channel tropism” network of GQWTF. The diamond and circular nodes represent the herbs and channel tropism, respectively. The node size is proportional to the node degree

We used the channel tropism of each herb to construct a herb-channel tropism network, as shown in Fig. 2. The organ affinity of the GQWTF herbs was highest for the liver, with the largest degree value of 4, followed by the kidney, lung, heart, and stomach in the network, with degree values of 3, 2, 1, and 1, respectively.

### LC–MS analysis of GQWTF

**Fig. 3:**
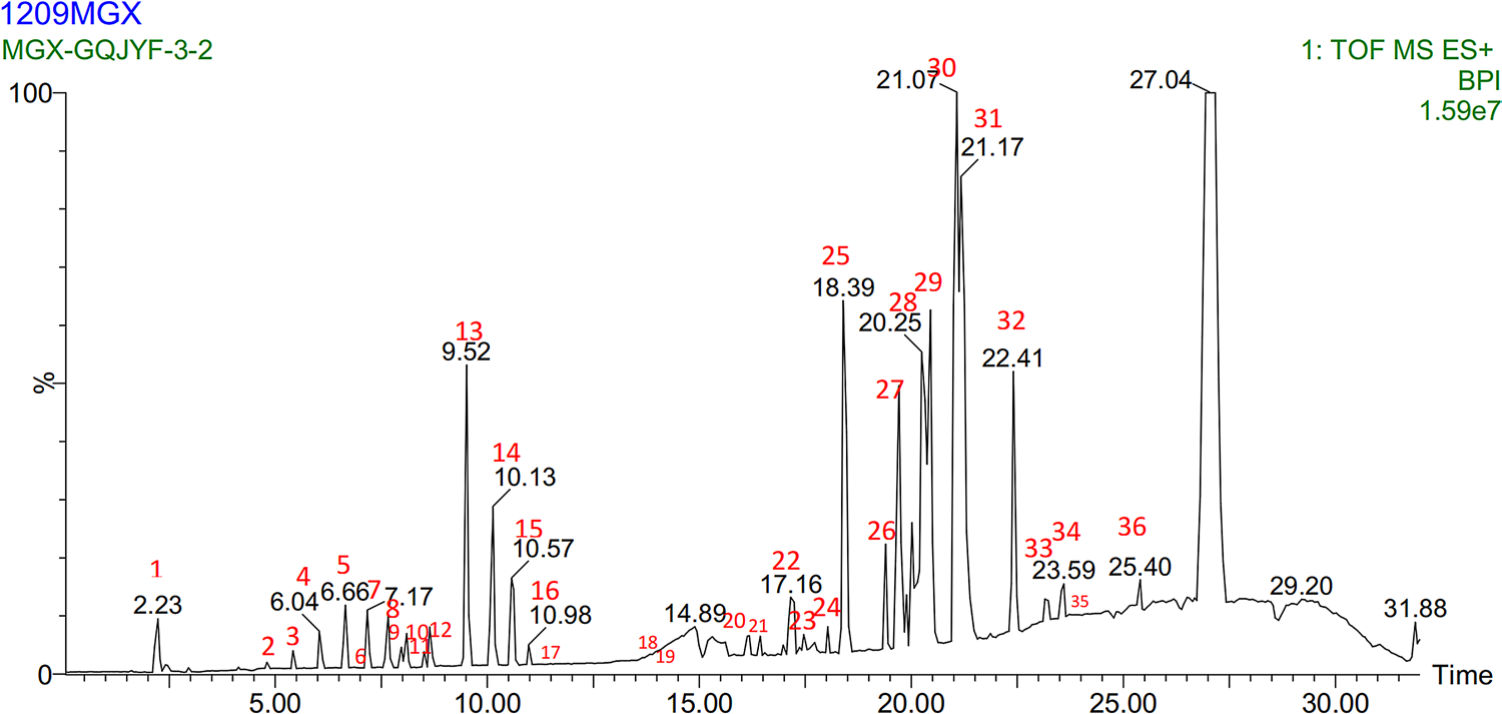
Peak intensity chromatograms of GQWTF acquired by LC–MS with 36 constituents labeled

Thirty-six compounds in GQWTF were identified by LC–MS (Additional file 1: Table S1), and Fig. 3 shows the peak intensity chromatograms. Compounds 1, 3, 4, 8, 9, 22, 23, 24, and 27 were unknown compounds, and compounds 28 and 29 were sulfur fumigation products of the herbs, which were discarded. After screening by ADME and related references [21–33], a total of 16 active ingredients were retained (Table 1).

**Table 1:** Active ingredients of GQWTF

No	Peak No.^a^	RT (min)	M+H	Product ion	Identification result	Chemical structure
1	2	4.804	354.2853	321.1312;305.1577;300.2020;283.1756	*N*-Methylcanadine	
2	5	6.632	453.2111	437.2361;432.2805;415.2533	Cyclo tetraleucyl	
3	6	7.134	903.4887; 957.5079	925.4880;741.4484	Timosaponin C	
4	7	7.173	497.2362	481.2635;476.3075	Oxypaeoniflorin	
5	10	8.125	585.2885	569.3146;564.3591;547.3326	Benzoylpaeoniflorin	
6	17	11.522	969.5364	789.4799;461.3982;443.3825	Ginsenoside Rd	
7	18	13.864	1109.6099	1131.5977	Ginsenoside Rb1	
8	19	14.044	1101.5818	947.5421; 785.4781; 461.3992	Ginsenoside Rc	
9	20	16.13	343.2959	365.2782; 250.1779;240.2325	1-*O*-caffeoyl glucoside	
10	21	16.54	314.1746	286.1446; 272.1521	Armepavine	
11	25	18.39	355.1047	259.1665;237.1842;124.0868	Chlorogenic acid	
12	26	19.707	463.2819	259.1099;243.1355	Lactiflorin	
13	30	21.097	579.2934	383.2043;301.1412	Timosaponin AI	
14	32	22.408	827.4404	684.2042;425.2144;301.1410	Esculentoside A	
15	33	23.258	331.2245	309.2423; 301.13	Iristectorigenin	
16	35	23.953	637.306	659.2884	Ginsenoside Rh4	

^**a**^Peak No. is consistent with the label of Fig. 3

### Compound-target network construction of GQWTF

**Fig. 4:**
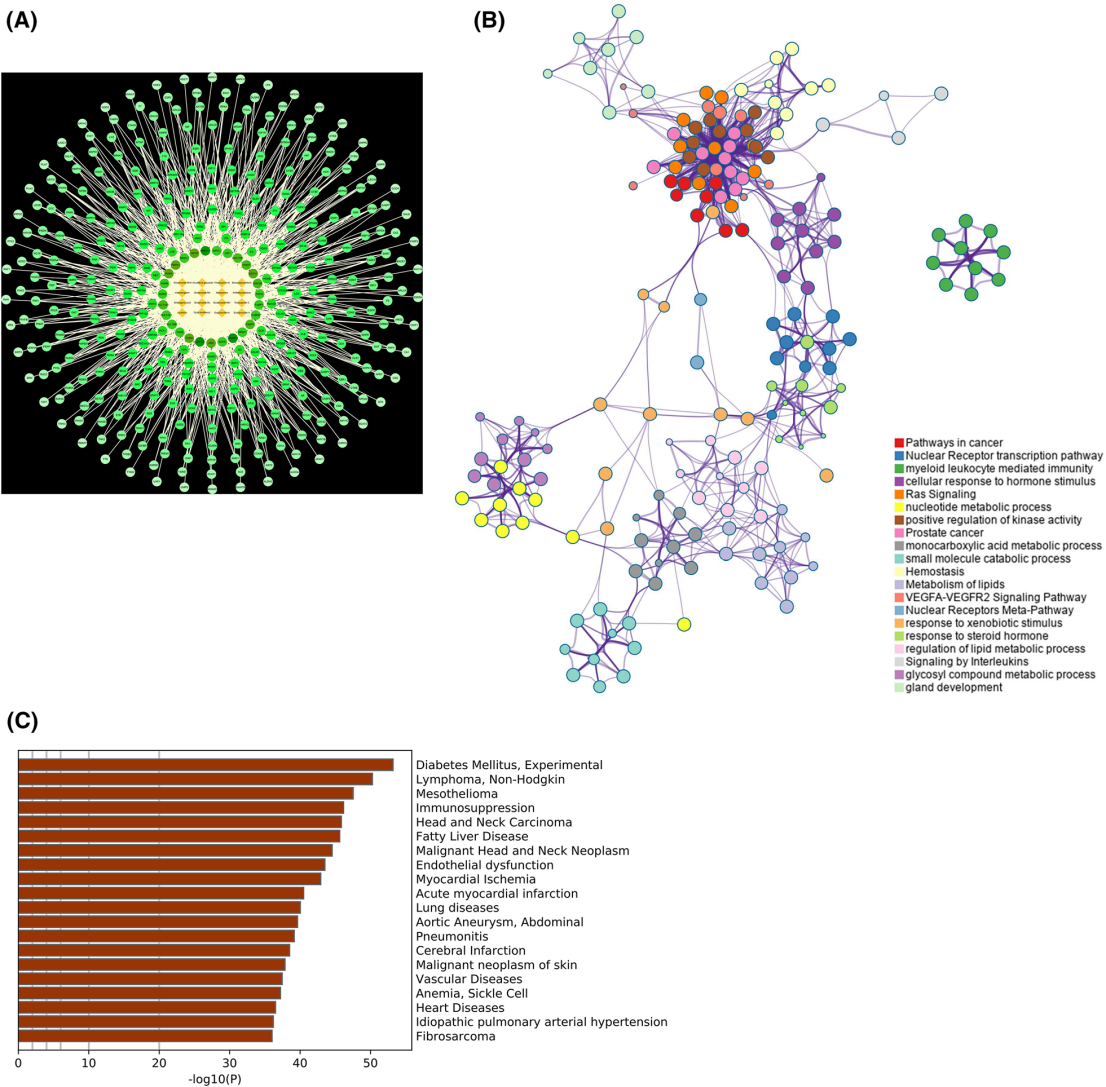
Network analysis of GQWTF. **A** ‘Compound -target’ network of GQWTF. Of which a link represents the interaction between a compound and a protein target node, rhombus indicates active compounds, and circle indicates target proteins. **B** Top 20 clusters with their representative enriched terms in pathway and process enrichment analysis for candidate targets of active ingredients in the GQWTF. Nodes were colored by cluster ID, where nodes that share the same cluster ID are typically close to each other. **C** Summary of enrichment analysis in DisGeNET

A total of 346 candidate targets of active ingredients in GQWTF were identified (Additional file 2: Table S2). We evaluated the relationships between these potential targets with active ingredients, and the herbs in the GQWTF with a constructed compound-target network (Fig. 4A), and the number of nodes and edges in this network were 362 and 1780, respectively.

The network indicated the potential relationship between compounds and targets, which implied the potential pharmacological mechanisms of GQWTF or the compounds. The nodes with the highest degree of connection to other compounds or targets represent hubs in the entire network, which are potential drugs or targets. For example, oxypaeoniflorin (degree = 214), benzoylpaeoniflorin (degree = 202), lactiflorin (degree = 192), timosaponin C (degree = 140), ginsenoside Rh4 (degree = 122), and 1-O-caffeoyl glucoside (degree = 121) were predicted to be important active compounds according to degree by topological analysis, indicating their critical roles in GQWTF. The targets of CRABP2 were linked to 14 compounds: oxypaeoniflorin, armepavine, benzoylpaeoniflorin, esculentoside A, ginsenoside Rb1, ginsenoside Rc, ginsenoside Rd, ginsenoside Rh4, lactiflorin, timosaponin C, timosaponin AI, *N*-methylcanadine, cyclotetraleucyl, and ristectorigenin. ZAP70 was linked to 14 compounds: oxypaeoniflorin, 1-O-caffeoyl glucoside, Benzoylpaeoniflorin, Chlorogenic acid, Esculentoside A, Ginsenoside Rb1, Ginsenoside Rc, Ginsenoside Rd, Ginsenoside Rh4, Lactiflorin, Timosaponin C, Timosaponin AI, Cyclo tetraleucyl, and Iristectorigenin. NR1I2, THRA, GSTA1, RARA, ABO, and PDPK1 were separately connected to 13 compounds, indicating that different compounds could target synergistically a single gene.

Subsequently, we performed pathway and process enrichment analysis for candidate targets of active ingredients in the GQWT. Statistically enriched terms were identified, and the top 20 clusters were visualised. To capture the relationships between the resulting terms, a subset of enriched terms was selected and converted into a network plot, where terms with a similarity > 0.3 are connected by edges. We selected the terms with the best *p*-values from each of the 20 clusters. The network is visualised using Cytoscape with “force-directed” layout, where each circle node represents an enriched term and is sized proportional to the number of input genes that fall into that term and coloured by its cluster identity. The selected terms from each cluster were shown as labels to have their term description (Fig. 4B), including Pathways in cancer, Nuclear Receptor transcription pathway, myeloid leukocyte mediated immunity, cellular response to hormone stimulus, Ras Signaling, nucleotide metabolic process, positive regulation of kinase activity, monocarboxylic acid metabolic process, small molecule catabolic process, Hemostasis, Metabolism of lipids, VEGFA-VEGFR2 Signaling Pathway, Nuclear Receptors Meta-Pathway, response to xenobiotic stimulus, response to steroid hormone, regulation of lipid metabolic process, Signaling by Interleukins, glycosyl compound metabolic process, etc.

Furthermore, using the DisGeNet database, the gene information related to the disease in candidate targets of active ingredients in the GQWTF were obtained, and the top 20 enriched clusters (one term per cluster) are shown in Fig. 4C. The most significant of which were Diabetes Mellitus, Experimental; while the other involved diseases were Lymphoma, Non-Hodgkin; Immunosuppression; Fatty Liver Disease; Endothelial dysfunction Vascular Diseases; Vascular Diseases; etc.

### GQWTF potential target-T2DM target network

**Fig. 5:**
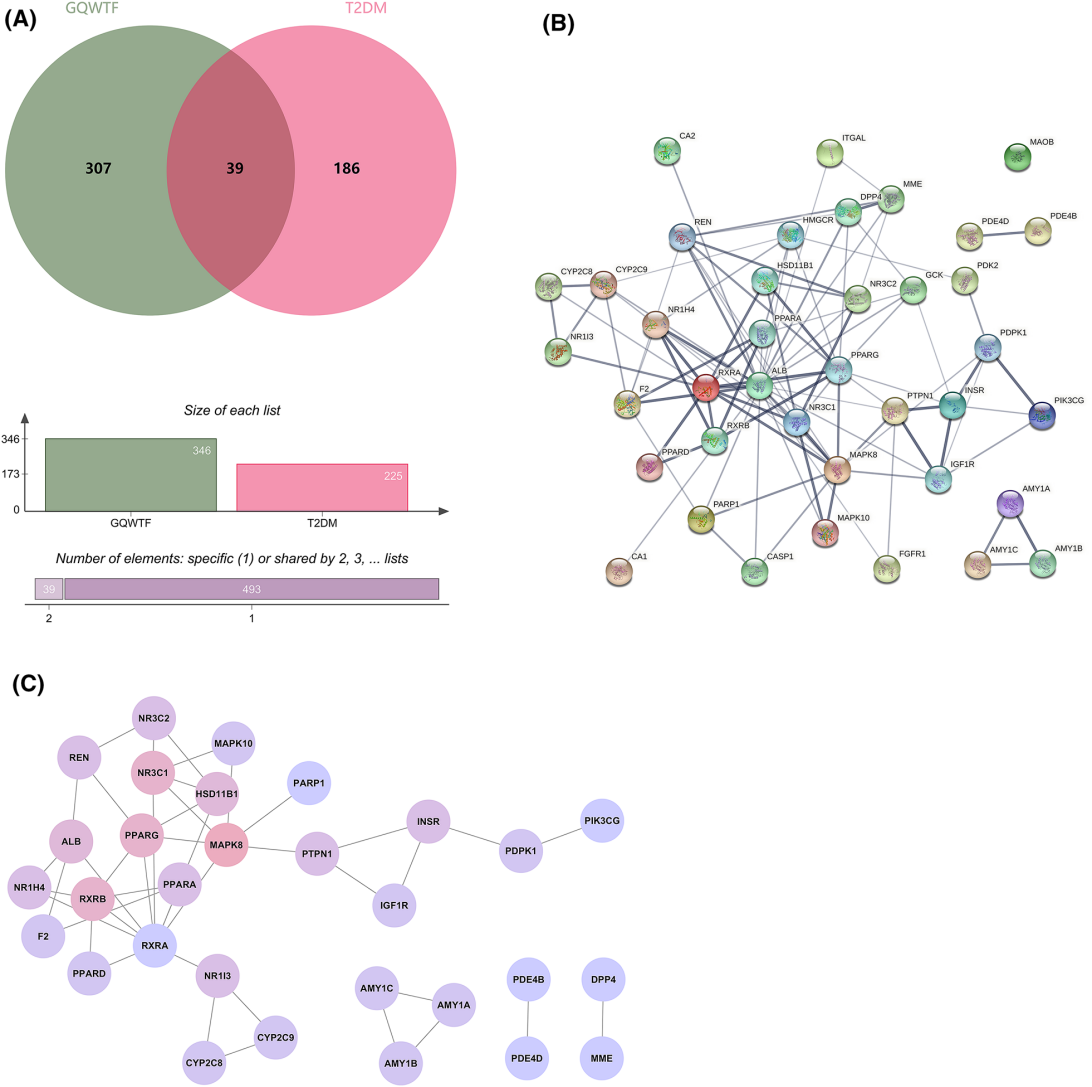
Overlapped targets analysis between GQWTF potential targets and T2DM targets. **A** Venn diagram of the target genes for GQWTF and T2DM. **B** Protein–protein interaction (PPI) network of all candidate targets of GQWTF for the treatment of T2DM by STRING. **C** PPI network of targets with high confidence scores (combined score ≥ 0.7)

For disease target identification, 153 and 92 target genes of “Type 2 Diabetes Mellitus” were identified from the Drugbank and TTD databases, respectively. Thus, we obtained 225 key nodes of target genes for disease targets after eliminating duplicates (Additional file 3: Table S3). Finally, a total of 39 targets were shared between 16 active ingredients of GQWTF-related targets and 225 T2DM-related targets (Fig. 5A, Table 2).

**Table 2:** Potential targets of GQWTF against T2DM

No	Protein name	Gene symbol	Uniprot ID
1	Bile acid receptor	NR1H4	Q96RI1
2	Caspase-1	CASP1	P29466
3	Cytochrome P450 2C8	CYP2C8	P10632
4	Cytochrome P450 2C9	CYP2C9	P11712
5	Hexokinase-4	GCK	P35557
6	cAMP-specific 3',5'-cyclic phosphodiesterase 4B	PDE4B	Q07343
7	Glucocorticoid receptor	NR3C1	P04150
8	Neprilysin	MME	P08473
9	Albumin	ALB	P02768
10	Peroxisome proliferator-activated receptor alpha	PPARA	Q07869
11	Renin	REN	P00797
12	[Pyruvate dehydrogenase (acetyl-transferring)] kinase isozyme 2, mitochondrial	PDK2	Q15119
13	Nuclear receptor subfamily 1 group I member 3	NR1I3	Q14994
14	Peroxisome proliferator-activated receptor delta	PPARD	Q03181
15	Mitogen-activated protein kinase 8	MAPK8	P45983
16	Tyrosine-protein phosphatase non-receptor type 1	PTPN1	P18031
17	Integrin alpha-L	ITGAL	P20701
18	3-hydroxy-3-methylglutaryl-coenzyme A reductase	HMGCR	P04035
19	cAMP-specific 3',5'-cyclic phosphodiesterase 4D	PDE4D	Q08499
20	Dipeptidyl peptidase 4	DPP4	P27487
21	Poly [ADP-ribose] polymerase 1	PARP1	P09874
22	Mineralocorticoid receptor	NR3C2	P08235
23	Insulin-like growth factor 1 receptor	IGF1R	P08069
24	3-phosphoinositide-dependent protein kinase 1	PDPK1	O15530
25	Corticosteroid 11-beta-dehydrogenase isozyme 1	HSD11B1	P28845
26	Fibroblast growth factor receptor 1	FGFR1	P11362
27	Retinoic acid receptor RXR-alpha	RXRA	P19793
28	Amine oxidase [flavin-containing] B	MAOB	P27338
29	Insulin receptor	INSR	P06213
30	Mitogen-activated protein kinase 10	MAPK10	P53779
31	Peroxisome proliferator-activated receptor gamma	PPARG	P37231
32	Carbonic anhydrase 2	CA2	P00918
33	Retinoic acid receptor RXR-beta	RXRB	P28702
34	Prothrombin	F2	P00734
35	Phosphatidylinositol 4,5-bisphosphate 3-kinase catalytic subunit gamma isoform	PIK3CG	P48736
36	Carbonic anhydrase 1	CA1	P00915
37	Alpha-amylase 1C	AMY1C	P0DTE8
38	Alpha-amylase 1A	AMY1A	P0DUB6
39	Alpha-amylase 1B	AMY1B	P0DTE7

The PPI relationships among these 39 target genes were generated from the STRING database, The PPI relationship network has 39 nodes and 98 edges, with an average of 5.03 node degree (Fig. 5B). Next, 30 shared targets with high confidence scores (combined score ≥ 0.7) (Fig. 5C), suggesting their strong interactions, were selected for further enrichment analysis.

### GO and pathway enrichment analysis

For GO analysis, we identified 58 biological processes, 4 cellular components, and 37 molecular functions with an adjusted *p* < 0.05. Results show that many biological processes are related to diabetes, such as cellular response to lipopolysaccharide, cellular response to insulin stimulus, bile acid and bile salt transport, carbohydrate metabolic process, digestion, neutrophil chemotaxis, insulin receptor signaling pathway, negative regulation of inflammatory response, negative regulation of sequestering of triglyceride, positive regulation of fatty acid oxidation, negative regulation of cholesterol storage, glucose homeostasis, and so on. GO analysis of cellular components showed that these processes occur mainly in nucleoplasm, receptor complex, membrane, and voltage-gated calcium channel complex.

**Fig. 6:**
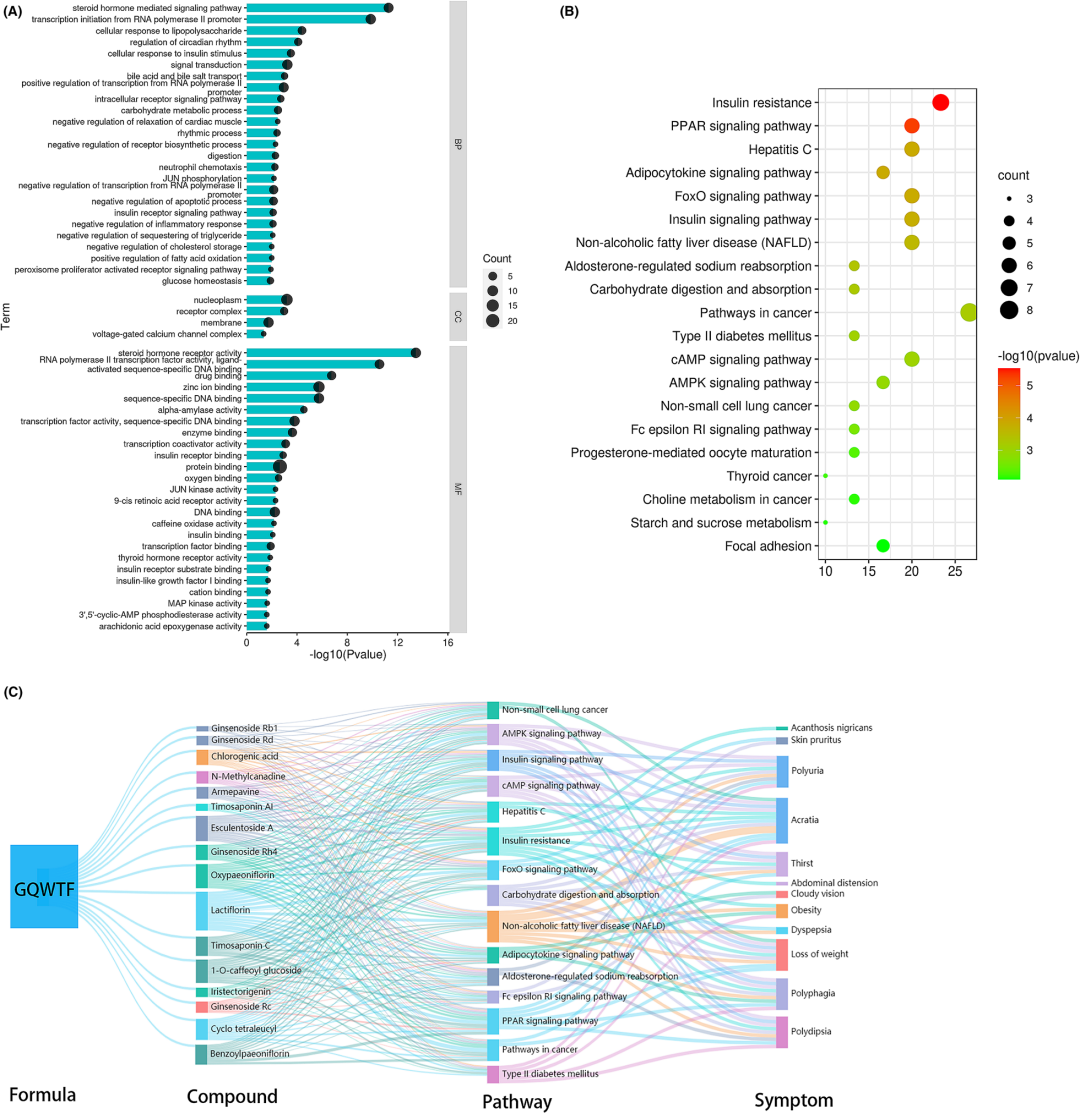
Enrichment analysis of the core targets from PPI analysis. **A** GO and **B** KEGG pathway enrichment analysis. **C** “Formula-compound-pathway-symptom” network

In addition to molecular functions, the enriched items were related to insulin receptor binding, protein binding, insulin binding, insulin receptor substrate binding, insulin-like growth factor I binding, MAP kinase activity, retinoid X receptor binding, insulin-like growth factor receptor binding, fatty acid binding, and so on. The top 25 GO items with the lowest *p*-value were shown in Fig. 6A, and details of all items are provided in the Additional file 4: Table S4.

KEGG pathway analysis was conducted to explore these targets further. These targets were highly enriched in Insulin resistance, PPAR signaling pathway, Adipocytokine signaling pathway, FoxO signaling pathway, Insulin signaling pathway, Aldosterone-regulated sodium reabsorption, Carbohydrate digestion and absorption, Type II diabetes mellitus, cAMP signaling pathway, AMPK signaling pathway, Fc epsilon RI signaling pathway, Starch and sucrose metabolism, and so on. The top 20 KEGG items with the lowest *p*-value were shown in Fig. 6B, and detailed information of all 35 pathways is provided in the Additional file 5: Table S5.

Furthermore, we searched the literature for the possible symptomatic improvement of these enriched pathways, such as thirst, polydipsia, acratia, loss of weight, polyphagia, polyuria, acanthosis nigricans, obesity, abdominal distension, cloudy vision, dyspepsia, and skin pruritus. We then created a network, which is shown in Fig. 6C.

### In vivo experimental validation

**Fig. 7:**
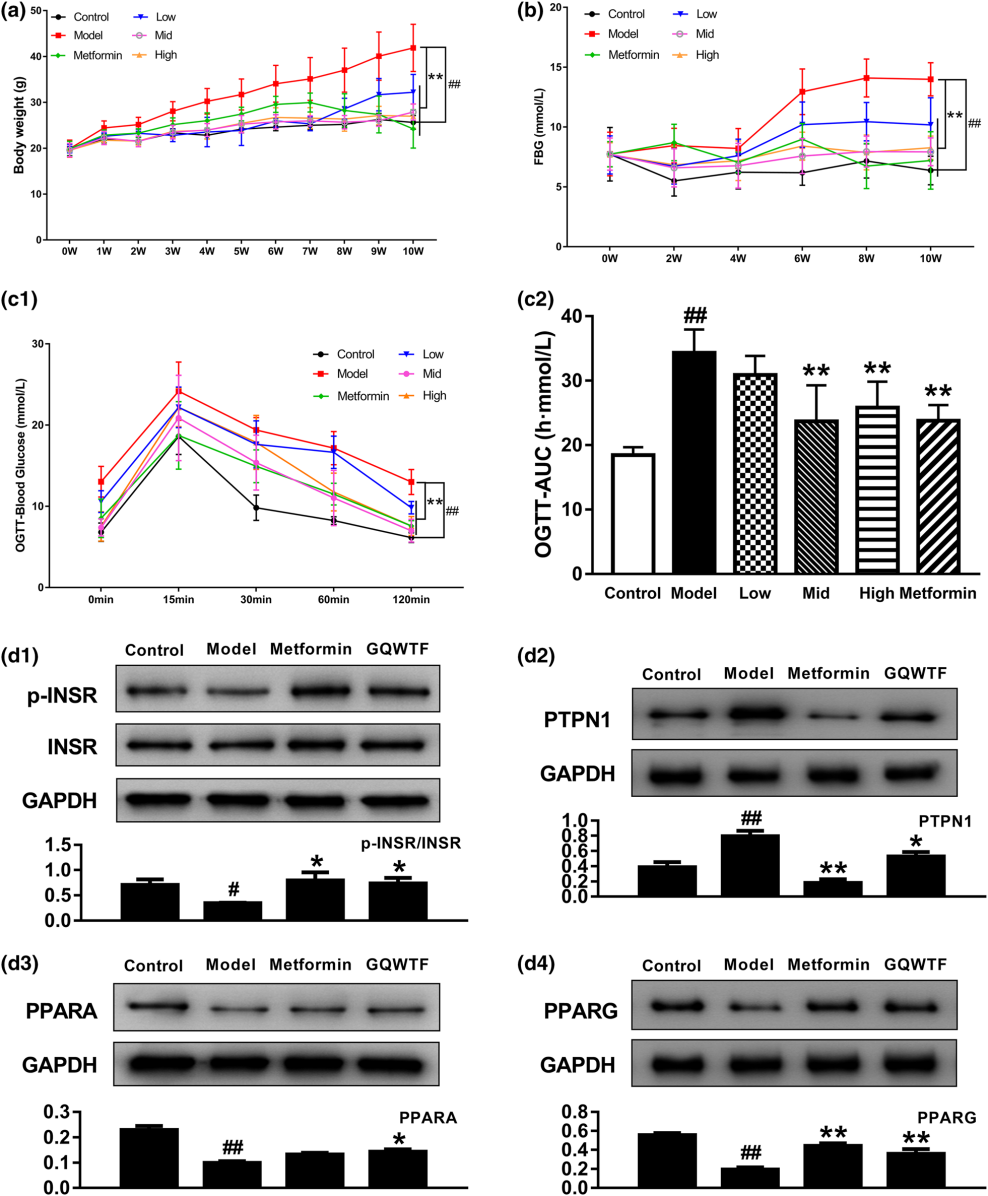
Effects of GQWTF on 10-week high-fat diet induced mice. Body weight **A** was monitored once a week, and fasting blood-glucose (FBG) **(B)** every 2 weeks during 10-week trial. **C** Dynamic changes of blood glucose during oral glucose tolerance test (OGTT), and AUC is area under the blood glucose-time curves of OGTT. **D** The relative protein expression of PTPN1, PPARA, PPARG and phosphorylation of INSR in liver using western blot analysis. Data are shown as means ± SEM of ten (**A**, **B**) or six (**C**) or three (**D**) independent experiments. ^#^*p* < 0.05, ^##^*p* < 0.01, compared with control group; *p < 0.05, **p < 0.01, compared with model group

During the 10-week treatment period, the weight of mice in the HFD group continued to increase and was significantly higher than that of the ND group at each time point. However, the weight of the mice significantly decreased in the drug-intervention groups (Fig. 7A). Similarly, the FBG levels were significantly higher in the HFD group than in the ND group. After the 10-week treatment period, GQWTF and metformin intervention achieved a significant glucose-lowering effect (Fig. 7B). The effects of GQWTF on reducing body weight and FBG levels showed good dose dependence.

OGTT is widely performed to evaluate whether the body is abnormally glucose tolerant and diabetic. The results showed that compared to the control group, the HFD-fed T2DM group had higher blood glucose levels during the 120 min period. GQWTF and metformin drug intervention also significantly reduced blood glucose during the test. Significant improvement in glucose clearance by drug treatment was also reflected by the lower peaks of blood glucose. Moreover, the AUC was calculated to further determine the improvement of glucose tolerance following long-term GQWTF treatment. The AUC in T2DM mice was much higher than that in control mice; meanwhile, the increased AUC in DM mice was reduced by GQWTF treatment (Fig. 7C).

Based on the pathway enrichment analysis, targets (INSR, PTPN1, PPARA, PPARG) from two of the most significant pathways, Insulin resistance, and PPAR signaling pathway, were selected for further experimental validation. As shown in Fig. 7D, western blot results showed that compared with the control group (normal diet), PTPN1 was upregulated in HFD-fed mice (*p* < 0.05), while treatment with GQWTF or metformin significantly inhibited the expression of PTPN1 (*p* < 0.01, *p* < 0.05). Treatment with drugs significantly blocked the inhibitory effect of HFD on INSR phosphorylation (*p* < 0.05). Furthermore, the overexpression of PPARA and PPARG induced by HFD was also abolished by treatment with GQWTF (*p* < 0.01, *p* < 0.05).

## Discussion

GQWTF is a proposed prescription for the prevention and treatment of T2DM, which is composed of GQZ, SY, ZM, CS, and XYS. As a holistic medicine, TCM is mainly based on the observation of the curative effect of empirical clinical practice, and its effectiveness depends on a variety of compounds, targets, and approaches. Thus, it is difficult to explore its mechanism of action. This study draws lessons from the research ideas of network pharmacology, combined with the theory of TCM, analytical chemistry, and molecular biology technology through the analysis of complex and multi-level interactions of various networks to identify multi-components and multi-targets involved in the prevention and treatment of T2DM by GQWTF.

The theory of channel tropism is an important part of the theory of TCM, which can reflect the attribution of TCM and prefers certain viscera, meridians, or specific parts. In the “herb-channel tropism” network of GQWTF, more herbs showed affinities for the liver, which indicates that GQWTF focuses more on treating diseases by recuperating the liver. As an important organ of systemic metabolism, the liver plays an important role in the development of insulin resistance and T2DM, related to hepatic fat accumulation and alterations in energy metabolism [34–36].

In this study, 16 potential active ingredients in GQWTF were obtained through qualitative analysis using LC–MS and ADME screening. Candidate targets of these active compounds in the GQWTF were identified by searching related database, thus “compound-target” network of GQWTF was built. The network topology parameter analysis results showed that Oxypaeoniflorin, Benzoylpaeoniflorin, Lactiflorin, Timosaponin C, Ginsenoside Rh4, and 1-O-caffeoyl glucoside, which had high degrees, were more important compounds in GQWTF. It also suggested that a single compound affects multiple targets, and some of the active ingredients in GQWTF could exert biological effects via multiple targets. Additionally, modern studies have also suggested the pharmacological activities of these compounds, such as hypoglycemic effect, anti-inflammation, anti-oxidation, and anti-tumour effects, [37–40] etc. The target linked to multiple compounds in the network also reflects the synergistic effect between different compounds. In brief, it can be seen that the multilevel and multi-target effects were jointly determined by multiple active compounds of GQWTF.

Furthermore, enrichment analysis of candidate targets of GQWTF revealed that it could exert a variety of pharmacological activities through multiple pathways, such as Pathways in cancer, VEGFA-VEGFR2 Signaling Pathway, regulation of lipid metabolic process, glycosyl compound metabolic process, Signaling by Interleukins, which reflects pharmacological activities such as anti-tumor, vascular regulation, glucose and lipid metabolism, and immune regulation, etc. As for the enrichment of these targets in the disease-related database, the most significant finding turned out to be Diabetes Mellitus and Experimental; thus, pharmacological prediction from the perspective of drugs alone has shown the potential of this prescription in the treatment of diabetes, which coincides with the theme of GQWTF in the treatment of T2DM in this study.

After retrieving T2DM related targets, 39 cross targets of GQWTF and T2DM were obtained, which were presumed to be putative targets of GQWTF for the treatment of T2DM. Moreover, we screened 30 core targets with stronger interactions through the PPI network for enrichment analysis. GO biological process enrichment analysis results showed that many biological processes of GQWTF in treating T2DM were closely involved in the key parts of the occurrence and development of diabetes mellitus, especially the top 25 items. For example, items related to inflammatory/immune response including cellular response to lipopolysaccharide, neutrophil chemotaxis, negative regulation of inflammatory response, and T2DM have been proven to be associated with chronic low-grade inflammation in a large number of studies [41–43]. Items related to insulin including cellular response to insulin stimulus, and insulin receptor signaling pathway were also involved. Insulin is the only hormone that can reduce blood glucose in the body. It cannot play its biological activity directly, as it needs to combine with insulin receptor on target organ cells to play its physiological role, and abnormalities in these physiological processes may induce the occurrence of T2DM [44, 45]. Items related to metabolism including bile acid and bile salt transport, carbohydrate metabolic process, digestion, negative regulation of sequestering of triglyceride, positive regulation of fatty acid oxidation, negative regulation of cholesterol storage, and glucose homeostasis were also involved. These biological processes also influence each other, especially the regulation of glucose and lipid metabolism, along with the whole process of the occurrence and development of T2DM [46–48]. Furthermore, the enriched items in molecular function analysis focused more on insulin, such as insulin receptor binding, insulin binding, insulin receptor substrate binding, insulin-like growth factor I binding, insulin-like growth factor receptor binding, etc.

Finally, KEGG pathway enrichment analysis was used to investigate the effect of GQWTF in the treatment of diabetes by regulating multiple signalling pathways. Of which Insulin resistance, a condition where cells become resistant to the effects of insulin and is often found in obesity, T2DM, non-alcoholic fatty liver disease, etc. [49–51]; for PPAR signaling pathway, which plays significantly in inflammation, insulin sensitivity, lipid metabolism, as well as the expression of hepatic enzymes regulating glucose homeostasis [52, 53]; for FoxO signaling pathway, which controls important physiological events, including apoptosis, glucose metabolism, oxidative stress resistance, by converting the external stimuli of insulin, growth factors, cytokines, and oxidative stress into cell-specific biological responses by regulating the transcriptional activity of target genes [54, 55]; for Fc epsilon RI signaling pathway, which contributes to inflammatory responses by regulating mediators and cytokines [56, 57]; there are also pathways that influence diabetes primarily by regulating metabolism, such as cAMP signaling pathway [58], AMPK signaling pathway [59], Starch and sucrose metabolism [60], Carbohydrate digestion and absorption [61], Aldosterone-regulated sodium reabsorption [62], Adipocytokine signaling pathway [63]; and other T2DM directly related pathways like Insulin signaling pathway, and Type II diabetes mellitus, are involved. In addition, combined with literature search, the corresponding symptoms of these pathways were synthesized to construct the "herbs-targets-pathways- symptoms" network, which represents a global view of GQWTF in treating T2DM, with their pathways that functions and related possible symptomatic improvement.

Finally, the efficacy of GQWTF was confirmed, and the key pathways were verified through animal experiments in vivo. The results showed that GQWTF reduced the body weight and blood glucose, which were abnormally elevated due to a high-fat diet. The abnormal glucose tolerance was also improved. In the validation of pathways, key targets (INSR, PTPN1, PPARA, and PPARG) from two of the most significant pathways in pathway enrichment analysis, Insulin resistance, and PPAR signaling pathway, were selected. The liver was selected according to the meridian network, which is also the key target tissue for insulin resistance in T2DM. INSR on hepatocyte surface would activate insulin receptor substrate (IRS) after binding with insulin, and then functionally mediate downstream like glucose/lipid metabolism, hepatocyte proliferation, DNA synthesis, and cell cycle regulation [64]. While PTPN1 dephosphorylates INSR and attenuates insulin signal transduction cascade, and human studies also demonstrated that PTPN1 expression increases in obese individuals and those with T2DM [65, 66]. For downstream PPARA and PPARG, which are important regulatory genes in cell differentiation and various metabolic processes, especially lipid and glucose metabolism [67, 68]. The regulatory effect of GQWTF on these key targets of these two pathways was verified by western blotting, the abnormal expression of proteins induced by high fat and the phosphorylation level of INSR were adjusted.

## Conclusions

In summary, we analysed the main components of GQWTF by LC–MS, and thus had a further understanding of its pharmacodynamic basis. By using the method of network pharmacology, the mechanism of GQWTF in the treatment of T2DM was analysed systematically at the molecular level, and the reliability of network pharmacology prediction was verified by animal experiments in vivo. Therefore, the potential mechanism of the synergistic action of GQWTF in the treatment of T2DM through multiple components, targets, and pathways was elaborated.

## Supplementary Information

**Additional file 1: Table S1.** Main compounds of GQWTF identified through LC-MS.

**Additional file 2: Table S2.** Candidate targets of active ingredients in GQWTF.

**Additional file 3: Table S3.** Candidate targets of T2DM.

**Additional file 4: Table S4-1.** The results of GO analysis-biological process (BP). **Table S4-2.** The results of GO analysis-cellular component (CC). **Table S4-3.** The results of GO analysis-molecular function (MF).

**Additional file 5: Table S5.** The results of KEGG analysis.

## Data Availability

The datasets used and/or analysed during the current study are available from the corresponding author on reasonable request.

## Abbreviations

T2DM: Type 2 diabetes mellitus
TCM: Traditional Chinese medicine
GQWTF: Gouqi-wentang formula
GQZ: Gou-Qi-Zi
SY: Sang-Ye
ZM: Zhi-Mu
CS: Chi-Shao
XYS: Xi-Yang-Shen
LC–MS: Liquid chromatography coupled with mass spectrometry
ADME: Absorption, distribution, metabolism, and excretion
TTD: Therapeutic Target Database
KEGG: Kyoto Encyclopedia of Genes and Genomes
GO: Gene Ontology
BP: Biological processes
PPI: Protein‒protein interaction
MF: Molecular functions
CC: Cellular components
HFD: High-fat diet
HED: Human equivalent dose
FBG: Fasting blood glucose
OGTT: Oral glucose tolerance test
AUC: Area under curve

## References

[1] Saeedi P, Salpea P, Karuranga S, Petersohn I, Malanda B, Gregg EW, Unwin N, Wild SH, Williams R (2020). Mortality attributable to diabetes in 20–79 years old adults, 2019 estimates: results from the International Diabetes Federation Diabetes Atlas, 9th edition. Diabetes Res Clin Pract..

[2] Neeland I, de Rocha Albuquerque N, Hughes C, Ayers C, Malloy C, Jin E (2020). Effects of empagliflozin treatment on glycerol-derived hepatic gluconeogenesis in adults with obesity: a randomized clinical trial. Obesity..

[3] Willey J, Wakefield M, Silver H (2020). Exploring the diets of adults with obesity and type II diabetes from nine diverse countries: dietary intakes, patterns, and quality. Nutrients.

[4] Saeedi P, Petersohn I, Salpea P, Malanda B, Karuranga S, Unwin N, Colagiuri S, Guariguata L, Motala A, Ogurtsova K, Shaw J, Bright D, Williams R (2019). Global and regional diabetes prevalence estimates for 2019 and projections for 2030 and 2045: results from the International Diabetes Federation Diabetes Atlas, 9 edition. Diabetes Res Clin Pract..

[5] Olveira G, Abuín J, López R, Herranz S, García-Almeida J, García-Malpartida K, Ferrer M, Cancer E, Luengo-Pérez L, Álvarez J, Aragón C, Ocón M, García-Manzanares Á, Bretón I, Serrano-Aguayo P, Pérez-Ferre N, López-Gómez J, Olivares J, Arraiza C, Tejera C, Martín J, García S, Abad Á, Alhambra M, Zugasti A, Parra J, Torrejón S, Tapia M (2020). Regular insulin added to total parenteral nutrition vs subcutaneous glargine in non-critically ill diabetic inpatients, a multicenter randomized clinical trial: INSUPAR trial. Clin Nutr.

[6] Tong X, Wu S, Lian F, Zhao M, Zhou S, Chen X, Yu B, Zhen Z, Qi L, Li P, Wang C, Sun H, Yuan C (2013). The safety and effectiveness of TM81, a Chinese herbal medicine, in the treatment of type 2 diabetes: a randomized double-blind placebo-controlled trial. Diabetes Obes Metab.

[7] Tian J, Lian F, Yang L, Tong X (2018). Evaluation of the Chinese herbal medicine Jinlida in type 2 diabetes patients based on stratification: results of subgroup analysis from a 12-week trial. J Diabetes.

[8] Hao S, Xu R, Li D, Zhu Z, Wang T, Liu K (2015). Attenuation of streptozotocin-induced lipid profile anomalies in the heart, brain, and mRNA expression of HMG-CoA reductase by diosgenin in rats. Cell Biochem Biophys.

[9] Masci A, Carradori S, Casadei MA, Paolicelli P, Petralito S, Ragno R, Cesa S (2018). *Lycium barbarum* polysaccharides: extraction, purification, structural characterisation and evidence about hypoglycaemic and hypolipidaemic effects. A review. Food Chem.

[10] Han X, Song C, Feng X, Wang Y, Meng T, Li S, Bai Y, Du B, Sun Q (2020). Isolation and hypoglycemic effects of water extracts from mulberry leaves in Northeast China. Food Funct.

[11] Zhang XP, Wang S, Li YH, Zhao D, An N, Wu JN, Zhang TT, Wu CM, Li YB (2015). Tadehaginoside modulates lipogenesis and glucose consumption in HepG2 cells. Nat Prod Res.

[12] Vuksan V, Xu ZZ, Jovanovski E, Jenkins AL, Beljan-Zdravkovic U, Sievenpiper JL, Mark Stavro P, Zurbau A, Duvnjak L, Li MZC (2019). Efficacy and safety of American ginseng (*Panax quinquefolius* L.) extract on glycemic control and cardiovascular risk factors in individuals with type 2 diabetes: a double-blind, randomized, cross-over clinical trial. Eur J Nutr..

[13] Juan YC, Tsai WJ, Lin YL, Wang GJ, Cheng JJ, Yang HY, Hsu CY, Liu HK (2010). The novel anti-hyperglycemic effect of *Paeoniae radix* via the transcriptional suppression of phosphoenopyruvate carboxykinase (PEPCK). Phytomedicine.

[14] Hopkins AL (2008). Network pharmacology: the next paradigm in drug discovery. Nat Chem Biol.

[15] Yuan H, Ma Q, Cui H, Liu G, Zhao X, Li W, Piao G (2017). How Can synergism of traditional medicines benefit from network pharmacology?. Molecules.

[16] Wang X, Shen Y, Wang S, Li S, Zhang W, Liu X, Lai L, Pei J, Li H (2017). PharmMapper 2017 update: a web server for potential drug target identification with a comprehensive target pharmacophore database. Nucleic Acids Res.

[17] Wishart DS, Knox C, Guo AC, Shrivastava S, Hassanali M, Stothard P, Chang Z, Woolsey J (2006). DrugBank: a comprehensive resource for in silico drug discovery and exploration. Nucleic Acids Res..

[18] Wang Y, Zhang S, Li F, Zhou Y, Zhang Y, Wang Z, Zhang R, Zhu J, Ren Y, Tan Y, Qin C, Li Y, Li X, Chen Y, Zhu F (2020). Therapeutic target database 2020: enriched resource for facilitating research and early development of targeted therapeutics. Nucleic Acids Res.

[19] Zhou Y, Zhou B, Pache L, Chang M, Khodabakhshi AH, Tanaseichuk O, Benner C, Chanda SK (2019). Metascape provides a biologist-oriented resource for the analysis of systems-level datasets. Nat Commun.

[20] Piñero J, Bravo À, Queralt-Rosinach N, Gutiérrez-Sacristán A, Deu-Pons J, Centeno E, García-García J, Sanz F, Furlong LI (2017). DisGeNET: a comprehensive platform integrating information on human disease-associated genes and variants. Nucleic Acids Res.

[21] Ji Y, Dou Y, Zhao Q, Zhang J, Yang Y, Wang T, Xia Y, Dai Y, Wei Z (2016). Paeoniflorin suppresses TGF-β mediated epithelial-mesenchymal transition in pulmonary fibrosis through a Smad-dependent pathway. Acta Pharmacologica Sinica..

[22] Jin Lee E, Chung HJ, Pyee Y, Hong JY, Joung Youn U, Seo EK, Kook LS (2014). Suppression of inducible nitric oxide synthase expression by nyasol and broussonin A, two phenolic compounds from *Anemarrhena asphodeloides*, through NF-κB transcriptional regulation in vitro and in vivo. Chem Biodivers.

[23] Yang Z, Weian C, Susu H, Hanmin W (2016). Protective effects of mangiferin on cerebral ischemia-reperfusion injury and its mechanisms. Eur J Pharmacol.

[24] Jiang S-Y, Li H, Tang J-J, Wang J, Luo J, Liu B, Wang J-K, Shi X-J, Cui H-W, Tang J, Yang F, Qi W, Qiu W-W, Song B-L (2018). Discovery of a potent HMG-CoA reductase degrader that eliminates statin-induced reductase accumulation and lowers cholesterol. Nat Commun.

[25] Zhou C, Wang X (2017). Rapid determination of isomeric benzoylpaeoniflorin and benzoylalbiflorin in rat plasma by LC-MS/MS Method. Int J Anal Chem.

[26] Zhou P, Zhang X, Guo M, Guo R, Wang L, Zhang Z, Lin Z, Dong M, Dai H, Ji X, Lu H (2019). Ginsenoside Rb1 ameliorates CKD-associated vascular calcification by inhibiting the Wnt/β-catenin pathway. J Cell Mol Med.

[27] Chang X, Jia H, Zhou C, Zhang H, Yu M, Yang J, Zou Z (2015). Role of Bai-Shao towards the antidepressant effect of Chaihu-Shu-Gan-San using metabonomics integrated with chemical fingerprinting. J Chromatogr B Analyt Technol Biomed Life Sci.

[28] Gao X, Li Y, Meng M, Wang P, Feng Y, Jia J, Qin X (2020). Exploration of chemical composition and absorption characteristics of Chaigui granules based on UHPLC-Q-orbitrap-MS/MS. J Pharm Biomed Anal..

[29] Sun J, Wu W, Guo Y, Qin Q, Liu S (2014). Pharmacokinetic study of ginsenoside Rc and simultaneous determination of its metabolites in rats using RRLC-Q-TOF-MS. J Pharm Biomed Anal.

[30] Liu JJ, Cheng Y, Shao YY, Chang ZP, Guo YT, Feng XJ, Xu D, Zhang JP, Song Y, Hou RG (2019). Comparative pharmacokinetics and metabolites study of seven major bioactive components of Shaoyao-Gancao decoction in normal and polycystic ovary syndrome rats by ultra high pressure liquid chromatography with tandem mass spectrometry. J Sep Sci.

[31] Jin B, Zhang C, Geng Y, Liu M (2020). Therapeutic effect of ginsenoside Rd on experimental autoimmune encephalomyelitis model mice: regulation of inflammation and Treg/Th17 Cell balance. Mediat Inflamm.

[32] de Souza P, da Silva LM, Boeing T, Somensi LB, Cechinel-Zanchett CC, Campos A, Krueger CMA, Bastos JK, Cechinel-Filho V, Andrade SF (2017). Influence of prostanoids in the diuretic and natriuretic effects of extracts and Kaempferitrin from *Bauhinia forficata* link leaves in rats. Phytother Res.

[33] Sun Z. Chemical composition and pharmacokinetics of Anemarrhenae Rhizoma Phellodendron. Master, Guangzhou University of traditional Chinese Medicine, 2017.

[34] Vangoitsenhoven R, Wilson R, Cherla D, Tu C, Kashyap S, Cummings D, Schauer P, Aminian A (2021). Presence of liver steatosis is associated with greater diabetes remission after gastric bypass surgery. Diabetes Care.

[35] Lu Y, Wang E, Chen Y, Zhou B, Zhao J, Xiang L, Qian Y, Jiang J, Zhao L, Xiong X, Lu Z, Wu D, Liu B, Yan J, Zhang R, Zhang H, Hu C, Li X (2020). Obesity-induced excess of 17-hydroxyprogesterone promotes hyperglycemia through activation of glucocorticoid receptor. J Clin Investig.

[36] Li J, Zhang Y, Ye Y, Li D, Liu Y, Lee E, Zhang M, Dai X, Zhang X, Wang S, Zhang J, Jia W, Zen K, Vidal-Puig A, Jiang X, Zhang C (2021). Pancreatic β cells control glucose homeostasis via the secretion of exosomal miR-29 family. J Extracell Vesicles..

[37] Zhang M, Feng L, Zhu M, Gu J, Wu C, Jia X (2013). Antioxidative and anti-inflammatory activities of paeoniflorin and oxypaeoniflora on AGEs-induced mesangial cell damage. Planta Medica..

[38] Zhu X, Fang Z-H (2014). New monoterpene glycosides from the root cortex of *Paeonia suffruticosa* and their potential anti-inflammatory activity. Nat Prod Res.

[39] Liu Y, Deng J, Fan D (2021). G-Rh4 improves pancreatic β-cells dysfunction in vivo and in vitro by increased expression of Nrf2 and its target genes. Food Chem Toxicol..

[40] Duan Z, Wei B, Deng J, Mi Y, Dong Y, Zhu C, Fu R, Qu L, Fan D (2018). The anti-tumor effect of ginsenoside Rh4 in MCF-7 breast cancer cells in vitro and in vivo. Biochem Biophys Res Commun.

[41] Prattichizzo F, De Nigris V, Spiga R, Mancuso E, La Sala L, Antonicelli R, Testa R, Procopio AD, Olivieri F, Ceriello A (2018). Inflammageing and metaflammation: the yin and yang of type 2 diabetes. Ageing Res Rev.

[42] Banerjee A, Singh J (2020). Remodeling adipose tissue inflammasome for type 2 diabetes mellitus treatment: current perspective and translational strategies. Bioeng Transl Med..

[43] Sen ZD, Danyeli LV, Woelfer M, Lamers F, Wagner G, Sobanski T, Walter M (2021). Linking atypical depression and insulin resistance-related disorders via low-grade chronic inflammation: Integrating the phenotypic, molecular and neuroanatomical dimensions. Brain Behav Immun.

[44] Fougerat A, Pan X, Smutova V, Heveker N, Cairo CW, Issad T, Larrivée B, Medin JA, Pshezhetsky AV (2018). Neuraminidase 1 activates insulin receptor and reverses insulin resistance in obese mice. Mol Metab.

[45] Wang Y, Zhou H, Palyha O, Mu J (2019). Restoration of insulin receptor improves diabetic phenotype in T2DM mice. JCI Insight..

[46] Sansome DJ, Xie C, Veedfald S, Horowitz M, Rayner CK, Wu T (2020). Mechanism of glucose-lowering by metformin in type 2 diabetes: role of bile acids. Diabetes Obes Metab.

[47] Xiang Z, Xie H, Tong Q, Pan J, Wan L, Fang J, Chen J (2021). Revealing hypoglycemic and hypolipidemic mechanism of Xiaokeyinshui extract combination on streptozotocin-induced diabetic mice in high sucrose/high fat diet by metabolomics and lipidomics. Biomed Pharmacother..

[48] Yuan X, Wang J, Yang S, Gao M, Cao L, Li X, Hong D, Tian S, Sun C (2020). Effect of the ketogenic diet on glycemic control, insulin resistance, and lipid metabolism in patients with T2DM: a systematic review and meta-analysis. Nutr Diabetes.

[49] Bao H, Liu Y, Zhang M, Chen Z, Zhang W, Ge Y, Kang D, Gao F, Shen Y (2021). Increased β-site APP cleaving enzyme 1-mediated insulin receptor cleavage in type 2 diabetes mellitus with cognitive impairment. Alzheimers Dement..

[50] Umano GR, Caprio S, Di Sessa A, Chalasani N, Dykas DJ, Pierpont B, Bale AE, Santoro N (2018). The rs626283 variant in the MBOAT7 gene is associated with insulin resistance and fatty liver in Caucasian obese youth. Am J Gastroenterol.

[51] Wu H, Ballantyne CM (2020). Metabolic inflammation and insulin resistance in obesity. Circ Res.

[52] Zhang C, Deng J, Liu D, Tuo X, Xiao L, Lai B, Yao Q, Liu J, Yang H, Wang N (2018). Nuciferine ameliorates hepatic steatosis in high-fat diet/streptozocin-induced diabetic mice through a PPARα/PPARγ coactivator-1α pathway. Br J Pharmacol.

[53] Xia X, Xu J, Wang X, Wang H, Lin Z, Shao K, Fang L, Zhang C, Zhao Y (2020). Jiaogulan tea (*Gpostemma pentaphyllum*) potentiates the antidiabetic effect of white tea via the AMPK and PI3K pathways in C57BL/6 mice. Food Funct.

[54] Kodani N, Nakae J (2020). Tissue-specific metabolic regulation of FOXO-binding protein: FOXO does not act alone. Cells.

[55] Nwadozi E, Roudier E, Rullman E, Tharmalingam S, Liu H, Gustafsson T, Haas TL (2016). Endothelial FoxO proteins impair insulin sensitivity and restrain muscle angiogenesis in response to a high-fat diet. FASEB J..

[56] Wang H-C, Huang S-K (2018). Metformin inhibits IgE- and aryl hydrocarbon receptor-mediated mast cell activation in vitro and in vivo. Eur J Immunol.

[57] Zhang X, Huang Q, Wang X, Deng Z, Li J, Yan X, Jauhiainen M, Metso J, Libby P, Liu J, Shi G-P (2019). Dietary cholesterol is essential to mast cell activation and associated obesity and diabetes in mice. Biochimica et Biophysica Acta..

[58] Chiefari E, Paonessa F, Iiritano S, Le Pera I, Palmieri D, Brunetti G, Lupo A, Colantuoni V, Foti D, Gulletta E, De Sarro G, Fusco A, Brunetti A (2009). The cAMP-HMGA1-RBP4 system: a novel biochemical pathway for modulating glucose homeostasis. BMC Biol.

[59] He Q, Wang L, Zhao R, Yan F, Sha S, Cui C, Song J, Hu H, Guo X, Yang M, Cui Y, Sun Y, Sun Z, Liu F, Dong M, Hou X, Chen L (2020). Mesenchymal stem cell-derived exosomes exert ameliorative effects in type 2 diabetes by improving hepatic glucose and lipid metabolism via enhancing autophagy. Stem Cell Res Ther.

[60] Song L, Liu H, Wang Y, Wang Y, Liu J, Zhou Z, Chu H, Zhuang P, Zhang Y (2015). Application of GC/MS-based metabonomic profiling in studying the therapeutic effects of Huangbai-Zhimu herb-pair (HZ) extract on streptozotocin-induced type 2 diabetes in mice. J Chromatogr B Analyt Technol Biomed Life Sci.

[61] Ansari P, Azam S, Hannan JMA, Flatt PR, Abdel Wahab YHA (2020). Anti-hyperglycaemic activity of H rosa-sinensis leaves is partly mediated by inhibition of carbohydrate digestion and absorption, and enhancement of insulin secretion. J Ethnopharmacol..

[62] Liu Y, Zhou L, Liu Z, Ma Y, Lin L, Zhu Y, Wang K, Li H (2020). Higher blood urea nitrogen and urinary calcium: new risk factors for diabetes mellitus in primary aldosteronism patients. Front Endocrinol.

[63] Komazaki R, Katagiri S, Takahashi H, Maekawa S, Shiba T, Takeuchi Y, Kitajima Y, Ohtsu A, Udagawa S, Sasaki N, Watanabe K, Sato N, Miyasaka N, Eguchi Y, Anzai K, Izumi Y (2017). Periodontal pathogenic bacteria, *Aggregatibacter actinomycetemcomitans* affect non-alcoholic fatty liver disease by altering gut microbiota and glucose metabolism. Sci Rep.

[64] Luo P, Zheng M, Zhang R, Zhang H, Liu Y, Li W, Sun X, Yu Q, Tipoe GL, Xiao J (2021). S-Allylmercaptocysteine improves alcoholic liver disease partly through a direct modulation of insulin receptor signaling. Acta Pharmaceutica Sinica B.

[65] Meshkani R, Taghikhani M, Al-Kateb H, Larijani B, Khatami S, Sidiropoulos GK, Hegele RA, Adeli K (2007). Polymorphisms within the protein tyrosine phosphatase 1B (PTPN1) gene promoter: functional characterization and association with type 2 diabetes and related metabolic traits. Clin Chem.

[66] Florez JC, Agapakis CM, Burtt NP, Sun M, Almgren P, Råstam L, Tuomi T, Gaudet D, Hudson TJ, Daly MJ, Ardlie KG, Hirschhorn JN, Groop L, Altshuler D (2005). Association testing of the protein tyrosine phosphatase 1B Gene (PTPN1) with type 2 diabetes in 7,883 people. Diabetes.

[67] Hong F, Xu P, Zhai Y (2018). The opportunities and challenges of peroxisome proliferator-activated receptors ligands in clinical drug discovery and development. Int J Mol Sci.

[68] Tan CK, Zhuang Y, Wahli W (2017). Synthetic and natural peroxisome proliferator-activated receptor (PPAR) agonists as candidates for the therapy of the metabolic syndrome. Expert Opin Ther Targets.

